# Supplementary material for: Mapping the evolution of fertility support policies in China: A content and instrumental analysis
Source: PLoS One. 2025 Oct 9;20(10):e0332137. doi: 10.1371/journal.pone.0332137 (PMC12510515; doi:10.1371/journal.pone.0332137)
Supplement: S1 Appendix — (ZIP) [file pone.0332137.s001.zip › S1 Appendix. 226 original policy documents/7-国家人口和计划生育委员会关于推动流动人口服务管理体制创新促进长江三角洲地区一体化发展的指导意见(FBM-CLI-4-221113).doc]

国家人口和计划生育委员会关于推动流动人口服务管理体制创新促进长江三角洲地区一体化发展的指导意见

**发布部门：** [国家人口和计划生育委员会(含国家计划生育委员会)(已撤销)](https://www.pkulaw.com/law/chl?Aggs.IssueDepartment=60304) [机构沿革](javascript:void(0))

**发文字号：**国人口发〔2009〕45号

**发布日期：**2009.05.31

**实施日期：**2009.05.31

**时效性：** [失效](https://www.pkulaw.com/law/chl?Aggs.TimelinessDic=02)

**效力级别：** [部门工作文件](https://www.pkulaw.com/law/chl?Aggs.EffectivenessDic=XE0303)

**法规类别：** [人口计划管理](https://www.pkulaw.com/law/chl?Aggs.Category=09701)

**专题分类：** [长三角一体化发展](https://www.pkulaw.com/law/chl?Aggs.SpecialType=024)

**失效依据：**[国家卫生健康委员会关于宣布失效第三批委文件的决定](javascript:TMP(316932,0,'chl'))

国家人口和计划生育委员会关于推动流动人口服务管理体制创新促进长江三角洲地区一体化发展的指导意见
(国人口发[2009]45号)

各省、自治区、直辖市人口计生委，计划单列市、新疆生产建设兵团人口计生委，解放军、武警部队人口和计划生育领导小组办公室，中直机关、中央国家机关人口计生委，委机关各单位，各直属、联系单位：
　　为贯彻落实国务院《[关于进一步推进长江三角洲地区改革开放和经济社会发展的指导意见](https://www.pkulaw.com/chl/674dfb796a098ad8bdfb.html)》（国发〔2008〕30号），加强外来人口服务和管理，建立健全区域内流动人口管理与服务协调机制，进一步推进基本公共服务均等化，推动区域流动人口服务管理体制机制创新，引导人口有序流动和合理分布，促进长三角地区率先实现经济社会一体化发展，提出以下意见：
　　**一、**推动流动人口服务管理体制创新，促进长三角地区一体化的重要意义
　　长江三角洲地区是我国综合实力最强的区域，也是重要的流动人口集聚区，在社会主义现代化建设全局中具有重要战略地位和带动作用。改革开放特别是推进上海浦东开发开放以来，长三角地区发展利用得天独厚的区位条件，在经济全球化和国际产业分工变动过程中，充分吸纳就业人口和促进人口集聚，先进制造业和现代服务业相互促进，人口与经济、社会、资源、环境协调发展取得巨大成就。今后较长时期，随着农村和中西部地区的人口继续向发达地区转移，长三角地区仍将是国内人口流动迁移的一个重要目的地。
　　流动人口服务管理工作事关稳定低生育水平、提高人口素质、改善人口结构、优化人口布局、保障人口安全、促进人口大国向人力资本强国转变战略目标的实现，是当前和今后一个时期统筹解决人口问题的一项重要任务。近年来，两省一市以科学发展观为统领，进一步深化合作内容、完善合作机制，积极推动流动人口服务管理体制创新，取得了显著成效。人口计生部门加强人口和计划生育工作的联动与合作，共同开展了“沪苏浙三省（市）人口发展战略研究”，出台了《关于加强流动人口计划生育服务和管理区域联动有关问题的意见》，启动了三省（市）人口信息数据共享工作，为推动流动人口计划生育区域“一盘棋”积累了经验。
　　党的十七届三中全会明确要求推动流动人口服务和管理体制创新。推动长三角地区流动人口服务管理体制创新，有利于深化流动人口计划生育区域协作，发挥长三角地区辐射和示范作用，加快形成全国“一盘棋”格局；有利于推进长三角地区以大城市化和大都市圈（群）为核心的城市化建设，实现长三角内涵优化和城市体系的有机联系，推进城乡、区域一体化发展；有利于引导人口有序流动和合理分布，实现产业布局与人口分布的相互促进，统筹解决人口问题，促进人口长期均衡发展，促进人口与经济、社会、资源、环境协调和可持续发展。长三角地区要切实按照党的十七届三中全会提出的推动流动人口服务和管理体制创新的要求，大胆实践，大力创新流动人口服务管理体制机制，促进长三角地区一体化发展。

　　**二、**指导思想与目标
　　（一）指导思想。以邓小平理论和“三个代表”重要思想为指导，深入贯彻落实科学发展观，按照构建社会主义和谐社会、统筹城乡和区域协调发展、推进基本公共服务均等化的要求，立足加快产业结构调整和提高区域经济整体竞争力，以体制改革和制度创新为核心，着力建立引导人口有序流动和合理分布的政策制度，加快推进长江三角洲地区一体化进程，促进人口与经济、社会、资源、环境的协调和可持续发展。
　　（二）近期目标。2009年，在两省一市实现省内“一盘棋”的基础上，长三角地区率先建立流动人口计划生育服务管理区域“一盘棋”格局，达成区域协作机制，实现流动人口计划生育基本项目免费技术服务全覆盖，积极推进区域人口信息化联动发展，为推动全国区域协调发展提供借鉴经验和模式。到2010年，区域协作机制进一步完善，根据人口流向进一步拓展协作范围，探索形成一套比较完整的区域协作、联动发展的规划、制度和措施；构建区域一体化的全员人口信息资源共享平台，实现流动人口信息实时变动、异地查询、跟踪管理和动态监测。
　　（三）“十二五”目标。到“十二五”末，结合户籍制度改革，基本实现以居住证为主的人口属地化管理制度，健全区域内流动人口统一管理、优质服务长效机制；加强计划生育服务管理，解决好流动人口就业、就医、定居、社会保障及子女教育等问题，率先实现长三角区域常住人口基本公共服务均等化；人口素质明显提高，人口有序流动和合理分布的格局初步显现，为长三角地区科学发展、率先发展、和谐发展创造良好的人口环境。

　　**三、**基本原则
　　（一）以人为本，注重民生。依据现有人口和计划生育法律法规和政策规定，按照政府职能转变和建设服务型政府的要求，重点解决两省一市间流动人口在享受基本公共服务和办理行政事务方面的问题，完善统筹解决人口问题的体制机制。
　　（二）先易后难，分步实施。按照两省一市《人口与计划生育条例》规定，依法履行部门职责，从解决群众迫切需要解决的问题入手，通过试点先行，示范带动，逐步完善流动人口服务管理体系。
　　（三）大胆创新，联动发展。探索运用信息化手段创新流动人口服务管理模式，推进流动人口服务管理信息的及时互动，提高管理效率，降低管理成本，为长三角地区一体化联动发展提供基础性决策支持信息，为区域一体化发展夯实基础。
　　（四）互惠互利，多方共赢。加强两省一市人口计生部门间的工作联动，通过三方密切协作，提高工作效率，稳步推进社会参与，真正使三地群众得到便利和实惠，形成互惠互利、多方共赢的局面。

　　**四、**创新体制、推进区域一体化的10项重点任务
　　（一）深化区域人口发展战略研究。立足长三角人口发展战略和人口发展功能区规划，整合两省一市研究力量，组建由人口学、经济学、社会学等多学科专家组成的咨询委员会，设立长三角人口流动迁移研究中心，建立公众参与、专家论证和政府决策相结合的长效研究机制。开展流动人口基础性、前瞻性研究，根据长三角产业布局和网络化城镇体系规划，正确把握户籍新政、土地制度、金融危机影响下的长三角人口流动迁移变动趋势，分析制约人口发展的体制机制障碍和政策措施，提出可借鉴性和可操作性的对策建议。编制长三角人口流动迁移区域规划，引导产业、人口有序转移和集聚，促进各要素的流通，进一步优化空间布局，为长三角地区经济社会又好又快发展提供理论支撑和智力支持。
　　（二）推进长三角人口与计划生育立法衔接工作。认真贯彻落实《[流动人口计划生育工作条例](https://www.pkulaw.com/chl/f6b2c35d55b7a40ebdfb.html)》，推动流动人口服务管理的规范化、制度化。以长三角改革区域户籍制度、推行以居住证为主的属地化管理制度为契机，开展人口政策调研，积极推动两省一市人口计生条例和相关政策的协调统一，制定流动人口计划生育服务管理关系转移、衔接的政策措施，加快推动长三角地区计划生育法制体系的相对统一。
　　（三）改善和保障计划生育民生问题。不断完善政府为主、社会补充的人口计生利益导向政策体系，逐步为符合条件的流动人口提供与户籍人口同等的计划生育保险以及生活、生育、生产等方面的帮扶奖励，流入地与流出地要协调做好奖励扶助的审核、备案和转移对接工作。加强与相关部门的统筹协调，促进人口计生与其他公共政策的衔接，在编制土地利用规划、城市发展规划、制定公共政策、建设公用设施等方面统筹考虑，让长期在城市就业、生活和居住的流动人口计划生育家庭享受民生保障政策，积极探索就业培训、养老保障、医疗服务、子女受教育等方面的联动转移制度。
　　（四）推进人口计生基本公共服务均等化。逐步打破行政区划限制，制定长三角流动人口基本公共服务均等化的标准和规范。全面落实流动人口计划生育免费技术服务，为符合条件的流动人口提供与户籍人口同等的计划生育、优生优育、生殖健康、避孕药具和技术服务，率先实现人口和计划生育优质服务基本覆盖的目标。
　　（五）积极探索城乡统一的人口登记制度。加强与相关部门的协调与沟通，结合户籍管理制度改革，整合居住证、暂住证和婚育证明等管理制度，探索实行城乡统一的人口属地登记制度，健全出生人口登记统计制度，探索推行出生登记实名制度。按照全国人口发展功能区规划，逐步地、有条件地解决长期在城市就业和居住的流动人口户籍问题，逐步破除城乡二元社会管理体制。
　　（六）构建流动人口信息化管理体系。积极构建区域一体化的流动人口信息化管理平台，建立以人口发展为中心、辅助政府管理决策的长三角区域人口信息中心。推动PADIS流动人口子系统的推广应用，按照“适时采集、动态监测、综合分析、科学决策”的目标，逐步完善流动人口信息管理系统。探索建立全员流动人口统计制度和重点地区流动人口统计监测体系，适时掌握流动人口变动状况及趋势，加强调查统计数据综合分析，逐步实现信息实时变动、异地查询、跟踪管理和动态监测。充分整合统计、公安、劳动保障、卫生、人口计生等部门资源，探索流动人口信息资源共享机制，为引导人口有序流动和合理分布提供信息支持。
　　（七）充分发挥宣传教育的引领作用。大力弘扬新型人口文化，不断创新整合两省一市宣传教育渠道和载体，强化社区宣传服务功能，深化新家庭计划和婚育新风进万家活动，将人口计生法律、法规、政策和科普知识的宣传教育覆盖到广大流动人口。创作更多反映流动人口现实生活的优秀人口文化产品，丰富流动人口的精神文化生活，引导他们树立科学、文明、进步的婚育观念，自觉地实行计划生育。
　　（八）实施人口计生服务管理便民利民工程。简化和规范计划生育事务办理程序，规范行政审批。进一步简化区域内流动人口包括两地婚姻生育手续的办理程序，规范《独生子女父母光荣证》发放，落实流动人口计划生育奖励待遇，协调做好《婚育证明》办理。
　　（九）切实维护流动人口合法权益。坚持依法行政，切实维护流动人口合法权益。加强监督检查，推行人口计生政务公开，建立公开、透明的行政体制和问责机制，实行投诉制、评估制、公示制和监察制。落实便民维权十项措施，发挥“12356”阳光计生服务热线作用，进一步畅通和拓宽流动人口维权渠道。完善区域内流动人口社会抚养费征收协作机制，加大联合打击“两非”行为的力度，重点推动省际边界地区的协作。
　　（十）坚持在体制改革上先行先试。研究制定流动人口服务管理体制改革试点方案，在国家综合配套改革试点区上海市浦东新区和江苏省无锡市、浙江省嘉兴市等地区先行先试，抓紧启动探索不同层级的服务管理体制机制新模式，培育典型，积累经验，提供典范，率先在重要领域和关键环节取得突破，带动全国流动人口服务管理体制机制创新。

　　**五、**保障措施
　　（一）建立统筹协调机制。国家人口计生委将长三角地区流动人口计划生育区域联动协作作为全国“一盘棋”“三年三步走”实施方案的率先突破口，予以重点部署、统筹协调、有序推进。两省一市人口计生部门要将这项工作列为“一把手工程”，切实做到一把手亲自抓、负总责。每年召开一次联席会议，由省（市）人口计生委主任轮流担任召集人，并落实联络员和日常联系制度，协调推进各项工作任务，促进长三角经济社会又好又快发展。
　　（二）建立投入保障机制。将流动人口计划生育服务管理纳入经常性工作范围，将流动人口服务管理经费纳入各级地方财政预算，实行分级管理、分级负担，并建立正常增长机制，确保流动人口服务管理有效运作。应根据流动人口的公共服务需要适当调整财力分配，加大对流动人口集中地区的财政转移支付力度，使政府财力与事权相匹配。
　　（三）健全考核评估机制。国家人口计生委根据“统筹管理、服务均等、信息共享、区域协作、双向考核”的要求，制定区域协作考核评估方案和标准，并组织开展督促检查和考核评估。两省一市应将区域联动协作纳入本行政区域人口计生工作目标管理责任制，明确要求，分解职责，分工协作，推动流动人口服务管理体制机制的创新，促进全国流动人口计划生育工作“一盘棋”格局的形成。

　　二○○九年五月三十一日

© 北大法宝：（[www.pkulaw.com](https://www.pkulaw.com/)) 专业提供法律信息、法学知识和法律软件领域各类解决方案。北大法宝为您提供丰富的参考资料，正式引用法规条文时请与标准文本核对。
欢迎查看所有[产品和服务](https://www.pkulaw.com/helps/2.html)。法宝快讯：[如何快速找到您需要的检索结果？](https://www.pkulaw.com/helps/70.html)[法宝](https://www.pkulaw.com/helps/69.html)V6有何新特色？

扫描二维码阅读原文

**原文链接：**<https://www.pkulaw.com/chl/eeacf8806e3736ebbdfb.html>
